# Diaqua­bis­(1-methyl-1*H*-imidazole-κ*N* ^3^)bis­[2-(naphthalen-1-yl)acetato-κ*O*]cobalt(II)

**DOI:** 10.1107/S1600536812013505

**Published:** 2012-04-04

**Authors:** Hong Zhao, Fu-Jun Yin, Xing-You Xu, Li-Jun Han

**Affiliations:** a Department of Chemical Engineering, Huaihai Institute of Technology, Lianyungang 222005, People’s Republic of China; b Jiangsu Marine Resources Development Research Institute, Huaihai Institute of Technology, Lianyungang 222005, People’s Republic of China; c Huaiyin Insititute of Technology, Huaiyin 223003, People’s Republic of China; d Department of Mathematics and Science, Huaihai Institute of Technology, Lianyungang 222005, People’s Republic of China

## Abstract

In the title compound, [Co(C_12_H_9_O_2_)_2_(C_4_H_6_N_2_)_2_(H_2_O)_2_], the Co^II^ ion is located on an inversion centre and displays a distorted octa­hedral coordination geometry. Two O atoms from two water mol­ecules and two carboxyl­ate O atoms from two 2-(naphthalen-1-yl)acetate ligands are in the equatorial plane and two N atoms from two 1-methyl-1*H*-imidazole ligands are in the axial positions. The structure is stabilized by intra­molecular O—H⋯O hydrogen bonds. Inter­molecular O—H⋯O hydrogen bonds link the complex mol­ecules into chains along [100].

## Related literature

For the structures of related complexes with 2-(naphthalen-1-yl)acetate ligands, see: Duan *et al.* (2007 ▶); Ji *et al.* (2011 ▶); Tang *et al.* (2006 ▶); Yang *et al.* (2008 ▶); Yin *et al.* (2011 ▶).
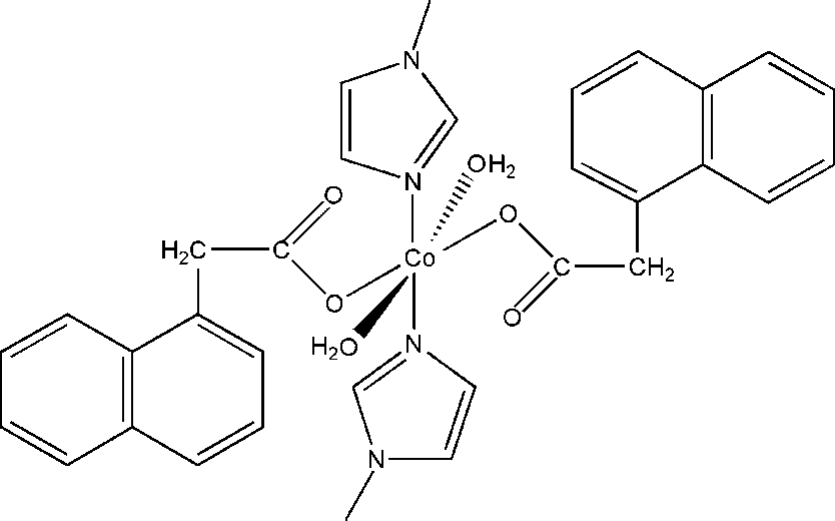


## Experimental

### 

#### Crystal data

[Co(C_12_H_9_O_2_)_2_(C_4_H_6_N_2_)_2_(H_2_O)_2_]
*M* *_r_* = 629.56
Monoclinic, 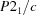

*a* = 7.3384 (7) Å
*b* = 24.582 (2) Å
*c* = 8.8559 (8) Å
β = 111.158 (1)°
*V* = 1489.8 (2) Å^3^
*Z* = 2
Mo *K*α radiation
μ = 0.63 mm^−1^
*T* = 298 K
0.34 × 0.30 × 0.20 mm

#### Data collection

Bruker APEXII CCD diffractometer
Absorption correction: multi-scan (*SADABS*; Sheldrick, 1996 ▶) *T*_min_ = 0.815, *T*_max_ = 0.885
13335 measured reflections
3356 independent reflections
2865 reflections with *I* > 2σ(*I*)
*R*_int_ = 0.024

#### Refinement

*R*[*F* ^2^ > 2σ(*F* ^2^)] = 0.032
*wR*(*F* ^2^) = 0.084
*S* = 1.05
3356 reflections
203 parameters
2 restraints
H atoms treated by a mixture of independent and constrained refinement
Δρ_max_ = 0.25 e Å^−3^
Δρ_min_ = −0.28 e Å^−3^

### 

Data collection: *APEX2* (Bruker, 2007 ▶); cell refinement: *SAINT* (Bruker, 2007 ▶); data reduction: *SAINT*; program(s) used to solve structure: *SHELXS97* (Sheldrick, 2008 ▶); program(s) used to refine structure: *SHELXL97* (Sheldrick, 2008 ▶); molecular graphics: *DIAMOND* (Brandenburg, 1999 ▶); software used to prepare material for publication: *SHELXTL* (Sheldrick, 2008 ▶).

## Supplementary Material

Crystal structure: contains datablock(s) I, global. DOI: 10.1107/S1600536812013505/hy2522sup1.cif

Structure factors: contains datablock(s) I. DOI: 10.1107/S1600536812013505/hy2522Isup2.hkl

Additional supplementary materials:  crystallographic information; 3D view; checkCIF report

## Figures and Tables

**Table 1:** Hydrogen-bond geometry (Å, °)

*D*—H⋯*A*	*D*—H	H⋯*A*	*D*⋯*A*	*D*—H⋯*A*
O3—H3*A*⋯O2^i^	0.84 (2)	2.13 (2)	2.8523 (18)	144 (2)
O3—H3*B*⋯O2	0.85 (2)	1.81 (2)	2.6463 (16)	168 (2)

Symmetry code: (i) 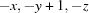.

**Fig. 1.**
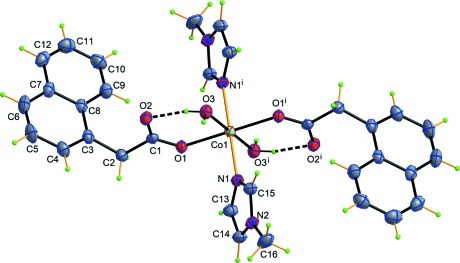
Molecular structure of the title compound. Displacement ellipsoids are drawn at the 30% probability level. Hydrogen bonds are shown as dashed lines. [Symmetry code: (i) -x+1, -y+1, -z.]

**Fig. 2.**
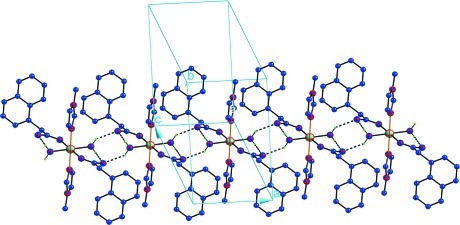
Part of the chain structure of the title compound. Hydrogen bonds are shown as dashed lines. H atoms not involved in hydrogen bonds are omitted for clarity.

## supplementary crystallographic information

### Comment

In recent years 2-(naphthalen-1-yl)acetate ligand has attracted many interests in coordination chemistry due to its ability to form metal complexes (Duan *et al.*, 2007; Ji *et al.*, 2011; Tang *et al.*, 2006; Yang *et al.*, 2008; Yin *et al.*, 2011). The crystal structure of the title compound was determined as part of an ongoing study of the properties of cobalt complexes containing imidazole ligands.

In the title compound (Fig. 1), the Co^II^ ion is located on an inversion centre and displays a distorted octahedral coordination geometry. Two O atoms from two water molecules and two carboxylate O atoms from two 2-(naphthalen-1-yl)acetate ligands are in the equatorial plane and two N atoms from two N-methylimidazole ligands are in the axial positions. The structure is stabilized by intramolecular O—H···O hydrogen bonds (Table 1). In the crystal, intermolecular O—H···O hydrogen bonds link the complex molecules into chains along [100] (Fig. 2).

### Experimental

The title compound was synthesized by the reaction of CoCl_2_.6H_2_O (71.3 mg, 0.3 mmol), 2-(naphthalen-1-yl)acetic acid (93 mg, 0.5 mmol), N-methylimidazole (32.8 mg, 0.4 mmol) and NaOH (20 mg, 0.5 mmol) in 15 ml of a water-ethanol mixture (v/v 1:1) under solvothermal conditions. The starting mixture was homogenized and transferred into a sealed Teflon-lined bomb (25 ml) and heated at 140° C for three days. After cooling, red crystals of the title compound were obtained, which were washed with distilled water and absolute ethyl alcohol (yield: 26.8% based on Co).

### Refinement

H atoms attached to C atoms were placed in calculated positions and refined as riding atoms, with C—H = 0.93 (CH), 0.97 (CH_2_) and 0.96 (CH_3_) Å and with *U*_iso_(H) = 1.2(1.5 for methyl)*U*_eq_(C). The water H atoms were located in a difference Fourier map and refined with a restraint of O—H = 0.85 (1) Å and with *U*_iso_(H) = 1.5*U*_eq_(O).

### Crystal data

**Table:**

[Co(C_12_H_9_O_2_)_2_(C_4_H_6_N_2_)_2_(H_2_O)_2_]	*F*(000) = 658
*M**_r_* = 629.56	*D*_x_ = 1.403 Mg m^−^^3^
Monoclinic, *P*2_1_/*c*	Mo *K*α radiation, λ = 0.71073 Å
Hall symbol: -P 2ybc	Cell parameters from 3356 reflections
*a* = 7.3384 (7) Å	θ = 2.6–27.3°
*b* = 24.582 (2) Å	µ = 0.63 mm^−^^1^
*c* = 8.8559 (8) Å	*T* = 298 K
β = 111.158 (1)°	Block, red
*V* = 1489.8 (2) Å^3^	0.34 × 0.30 × 0.20 mm
*Z* = 2	

### Data collection

**Table:**

Bruker APEXII CCD diffractometer	3356 independent reflections
Radiation source: fine-focus sealed tube	2865 reflections with *I* > 2σ(*I*)
Graphite monochromator	*R*_int_ = 0.024
φ and ω scans	θ_max_ = 27.3°, θ_min_ = 2.6°
Absorption correction: multi-scan (*SADABS*; Sheldrick, 1996)	*h* = −9→9
*T*_min_ = 0.815, *T*_max_ = 0.885	*k* = −31→30
13335 measured reflections	*l* = −11→11

### Refinement

**Table:**

Refinement on *F*^2^	Primary atom site location: structure-invariant direct methods
Least-squares matrix: full	Secondary atom site location: difference Fourier map
*R*[*F*^2^ > 2σ(*F*^2^)] = 0.032	Hydrogen site location: inferred from neighbouring sites
*wR*(*F*^2^) = 0.084	H atoms treated by a mixture of independent and constrained refinement
*S* = 1.05	*w* = 1/[σ^2^(*F*_o_^2^) + (0.0408*P*)^2^ + 0.3567*P*] where *P* = (*F*_o_^2^ + 2*F*_c_^2^)/3
3356 reflections	(Δ/σ)_max_ < 0.001
203 parameters	Δρ_max_ = 0.25 e Å^−^^3^
2 restraints	Δρ_min_ = −0.28 e Å^−^^3^

### Special details

**Table:**

Geometry. All esds (except the esd in the dihedral angle between two l.s. planes) are estimated using the full covariance matrix. The cell esds are taken into account individually in the estimation of esds in distances, angles and torsion angles; correlations between esds in cell parameters are only used when they are defined by crystal symmetry. An approximate (isotropic) treatment of cell esds is used for estimating esds involving l.s. planes.
Refinement. Refinement of F^2^ against ALL reflections. The weighted R-factor wR and goodness of fit S are based on F^2^, conventional R-factors R are based on F, with F set to zero for negative F^2^. The threshold expression of F^2^ > 2sigma(F^2^) is used only for calculating R-factors(gt) etc. and is not relevant to the choice of reflections for refinement. R-factors based on F^2^ are statistically about twice as large as those based on F, and R- factors based on ALL data will be even larger.

### Fractional atomic coordinates and isotropic or equivalent isotropic displacement parameters (Å^2^)

**Table:**

	*x*	*y*	*z*	*U*_iso_*/*U*_eq_	
Co1	0.5000	0.5000	0.0000	0.03250 (10)	
N1	0.3651 (2)	0.49725 (5)	−0.25228 (16)	0.0396 (3)	
N2	0.2163 (2)	0.46891 (6)	−0.50250 (16)	0.0439 (3)	
O1	0.37360 (16)	0.57809 (4)	0.00491 (14)	0.0417 (3)	
O2	0.14963 (16)	0.55623 (4)	0.11124 (14)	0.0429 (3)	
O3	0.26123 (16)	0.46019 (5)	0.04077 (14)	0.0414 (3)	
C1	0.2365 (2)	0.58909 (6)	0.05248 (17)	0.0344 (3)	
C2	0.1641 (3)	0.64816 (7)	0.0303 (2)	0.0430 (4)	
H2A	0.2760	0.6720	0.0518	0.052*	
H2B	0.0803	0.6533	−0.0819	0.052*	
C3	0.0536 (2)	0.66527 (6)	0.1363 (2)	0.0405 (4)	
C4	−0.1444 (3)	0.67227 (7)	0.0715 (2)	0.0508 (4)	
H4	−0.2105	0.6649	−0.0377	0.061*	
C5	−0.2506 (3)	0.69042 (8)	0.1662 (3)	0.0631 (6)	
H5	−0.3851	0.6951	0.1186	0.076*	
C6	−0.1594 (3)	0.70103 (8)	0.3253 (3)	0.0601 (5)	
H6	−0.2311	0.7136	0.3859	0.072*	
C7	0.0448 (3)	0.69323 (6)	0.4006 (2)	0.0471 (4)	
C8	0.1530 (2)	0.67521 (6)	0.3049 (2)	0.0393 (3)	
C9	0.3577 (3)	0.66855 (7)	0.3824 (2)	0.0494 (4)	
H9	0.4314	0.6567	0.3227	0.059*	
C10	0.4479 (3)	0.67927 (9)	0.5436 (3)	0.0636 (5)	
H10	0.5826	0.6751	0.5918	0.076*	
C11	0.3404 (4)	0.69649 (8)	0.6373 (3)	0.0686 (6)	
H11	0.4038	0.7034	0.7471	0.082*	
C12	0.1447 (4)	0.70302 (7)	0.5682 (3)	0.0611 (5)	
H12	0.0744	0.7141	0.6317	0.073*	
C13	0.3534 (3)	0.53921 (7)	−0.3573 (2)	0.0473 (4)	
H13	0.4010	0.5742	−0.3270	0.057*	
C14	0.2625 (3)	0.52220 (8)	−0.5115 (2)	0.0510 (4)	
H14	0.2367	0.5428	−0.6051	0.061*	
C15	0.2803 (2)	0.45573 (7)	−0.34511 (19)	0.0428 (4)	
H15	0.2666	0.4214	−0.3062	0.051*	
C16	0.1186 (3)	0.43256 (10)	−0.6383 (2)	0.0617 (5)	
H16A	0.2063	0.4239	−0.6932	0.093*	
H16B	0.0045	0.4502	−0.7122	0.093*	
H16C	0.0810	0.3997	−0.5986	0.093*	
H3A	0.161 (3)	0.4468 (11)	−0.029 (2)	0.093*	
H3B	0.222 (4)	0.4886 (7)	0.074 (3)	0.093*	

### Atomic displacement parameters (Å^2^)

**Table:**

	*U*^11^	*U*^22^	*U*^33^	*U*^12^	*U*^13^	*U*^23^
Co1	0.03202 (16)	0.03393 (16)	0.03224 (16)	0.00035 (11)	0.01242 (11)	−0.00172 (11)
N1	0.0412 (7)	0.0412 (7)	0.0347 (7)	0.0010 (6)	0.0119 (6)	0.0007 (6)
N2	0.0419 (7)	0.0557 (9)	0.0331 (7)	−0.0014 (6)	0.0124 (6)	−0.0032 (6)
O1	0.0423 (6)	0.0395 (6)	0.0479 (6)	0.0053 (5)	0.0220 (5)	0.0010 (5)
O2	0.0454 (6)	0.0376 (6)	0.0503 (7)	0.0003 (5)	0.0230 (5)	−0.0017 (5)
O3	0.0391 (6)	0.0393 (6)	0.0479 (7)	−0.0033 (5)	0.0183 (5)	−0.0046 (5)
C1	0.0346 (7)	0.0365 (8)	0.0287 (7)	−0.0001 (6)	0.0074 (6)	−0.0041 (6)
C2	0.0501 (9)	0.0376 (8)	0.0443 (9)	0.0055 (7)	0.0205 (7)	0.0026 (7)
C3	0.0441 (9)	0.0276 (7)	0.0532 (10)	0.0021 (6)	0.0218 (8)	0.0009 (6)
C4	0.0440 (9)	0.0412 (9)	0.0632 (12)	0.0005 (7)	0.0145 (8)	−0.0032 (8)
C5	0.0397 (10)	0.0559 (12)	0.0970 (17)	0.0036 (8)	0.0287 (11)	−0.0024 (11)
C6	0.0599 (12)	0.0485 (11)	0.0881 (16)	0.0029 (9)	0.0462 (12)	−0.0084 (10)
C7	0.0602 (11)	0.0293 (8)	0.0619 (11)	−0.0016 (7)	0.0343 (9)	−0.0039 (7)
C8	0.0457 (9)	0.0255 (7)	0.0511 (9)	−0.0002 (6)	0.0226 (7)	−0.0007 (6)
C9	0.0478 (10)	0.0444 (9)	0.0567 (11)	0.0011 (7)	0.0197 (8)	−0.0005 (8)
C10	0.0605 (12)	0.0554 (12)	0.0640 (13)	−0.0008 (9)	0.0094 (10)	0.0010 (10)
C11	0.0946 (17)	0.0521 (12)	0.0520 (12)	−0.0037 (11)	0.0180 (11)	−0.0053 (9)
C12	0.0958 (17)	0.0387 (10)	0.0614 (13)	−0.0015 (10)	0.0437 (12)	−0.0070 (9)
C13	0.0540 (10)	0.0429 (9)	0.0455 (10)	−0.0023 (8)	0.0186 (8)	0.0039 (7)
C14	0.0554 (11)	0.0575 (11)	0.0402 (9)	0.0035 (9)	0.0173 (8)	0.0105 (8)
C15	0.0467 (9)	0.0444 (9)	0.0364 (8)	−0.0006 (7)	0.0141 (7)	0.0014 (7)
C16	0.0622 (12)	0.0798 (14)	0.0396 (10)	−0.0100 (10)	0.0142 (9)	−0.0162 (9)

### Geometric parameters (Å, º)

**Table:**

Co1—N1	2.0928 (13)	C5—H5	0.9300
Co1—O1	2.1392 (11)	C6—C7	1.416 (3)
Co1—O3	2.1483 (11)	C6—H6	0.9300
N1—C15	1.317 (2)	C7—C12	1.419 (3)
N1—C13	1.371 (2)	C7—C8	1.425 (2)
N2—C15	1.340 (2)	C8—C9	1.418 (2)
N2—C14	1.363 (2)	C9—C10	1.364 (3)
N2—C16	1.461 (2)	C9—H9	0.9300
O1—C1	1.2528 (18)	C10—C11	1.401 (3)
O2—C1	1.2525 (18)	C10—H10	0.9300
O3—H3A	0.84 (2)	C11—C12	1.352 (3)
O3—H3B	0.85 (2)	C11—H11	0.9300
C1—C2	1.534 (2)	C12—H12	0.9300
C2—C3	1.505 (2)	C13—C14	1.352 (3)
C2—H2A	0.9700	C13—H13	0.9300
C2—H2B	0.9700	C14—H14	0.9300
C3—C4	1.367 (2)	C15—H15	0.9300
C3—C8	1.427 (2)	C16—H16A	0.9600
C4—C5	1.408 (3)	C16—H16B	0.9600
C4—H4	0.9300	C16—H16C	0.9600
C5—C6	1.349 (3)		
			
N1—Co1—N1^i^	180.000 (1)	C6—C5—C4	120.71 (18)
N1—Co1—O1	90.52 (5)	C6—C5—H5	119.6
N1^i^—Co1—O1	89.48 (5)	C4—C5—H5	119.6
N1—Co1—O1^i^	89.48 (5)	C5—C6—C7	120.51 (18)
N1^i^—Co1—O1^i^	90.52 (5)	C5—C6—H6	119.7
O1—Co1—O1^i^	180.0	C7—C6—H6	119.7
N1—Co1—O3^i^	86.31 (5)	C6—C7—C12	121.94 (18)
N1^i^—Co1—O3^i^	93.69 (5)	C6—C7—C8	118.93 (17)
O1—Co1—O3^i^	88.89 (4)	C12—C7—C8	119.13 (17)
O1^i^—Co1—O3^i^	91.11 (4)	C9—C8—C7	117.92 (16)
N1—Co1—O3	93.69 (5)	C9—C8—C3	122.62 (15)
N1^i^—Co1—O3	86.31 (5)	C7—C8—C3	119.46 (15)
O1—Co1—O3	91.11 (4)	C10—C9—C8	120.91 (18)
O1^i^—Co1—O3	88.89 (4)	C10—C9—H9	119.5
O3^i^—Co1—O3	180.00 (6)	C8—C9—H9	119.5
C15—N1—C13	105.06 (14)	C9—C10—C11	120.9 (2)
C15—N1—Co1	128.77 (11)	C9—C10—H10	119.6
C13—N1—Co1	126.13 (11)	C11—C10—H10	119.6
C15—N2—C14	106.99 (14)	C12—C11—C10	120.1 (2)
C15—N2—C16	126.28 (16)	C12—C11—H11	119.9
C14—N2—C16	126.72 (15)	C10—C11—H11	119.9
C1—O1—Co1	127.40 (10)	C11—C12—C7	121.06 (19)
Co1—O3—H3A	127.2 (18)	C11—C12—H12	119.5
Co1—O3—H3B	94.9 (19)	C7—C12—H12	119.5
H3A—O3—H3B	105 (3)	C14—C13—N1	109.88 (16)
O2—C1—O1	126.26 (14)	C14—C13—H13	125.1
O2—C1—C2	117.33 (13)	N1—C13—H13	125.1
O1—C1—C2	116.37 (14)	C13—C14—N2	106.28 (15)
C3—C2—C1	115.10 (13)	C13—C14—H14	126.9
C3—C2—H2A	108.5	N2—C14—H14	126.9
C1—C2—H2A	108.5	N1—C15—N2	111.79 (15)
C3—C2—H2B	108.5	N1—C15—H15	124.1
C1—C2—H2B	108.5	N2—C15—H15	124.1
H2A—C2—H2B	107.5	N2—C16—H16A	109.5
C4—C3—C8	118.82 (16)	N2—C16—H16B	109.5
C4—C3—C2	120.25 (16)	H16A—C16—H16B	109.5
C8—C3—C2	120.92 (14)	N2—C16—H16C	109.5
C3—C4—C5	121.53 (19)	H16A—C16—H16C	109.5
C3—C4—H4	119.2	H16B—C16—H16C	109.5
C5—C4—H4	119.2		
			
O1—Co1—N1—C15	−139.37 (14)	C6—C7—C8—C9	178.98 (16)
O1^i^—Co1—N1—C15	40.63 (14)	C12—C7—C8—C9	−0.8 (2)
O3^i^—Co1—N1—C15	131.77 (15)	C6—C7—C8—C3	−0.2 (2)
O3—Co1—N1—C15	−48.23 (15)	C12—C7—C8—C3	179.99 (15)
O1—Co1—N1—C13	43.28 (14)	C4—C3—C8—C9	179.47 (16)
O1^i^—Co1—N1—C13	−136.72 (14)	C2—C3—C8—C9	−1.4 (2)
O3^i^—Co1—N1—C13	−45.58 (14)	C4—C3—C8—C7	−1.3 (2)
O3—Co1—N1—C13	134.42 (14)	C2—C3—C8—C7	177.79 (14)
N1—Co1—O1—C1	107.01 (13)	C7—C8—C9—C10	−0.2 (3)
N1^i^—Co1—O1—C1	−72.99 (13)	C3—C8—C9—C10	178.98 (17)
O3^i^—Co1—O1—C1	−166.69 (12)	C8—C9—C10—C11	0.8 (3)
O3—Co1—O1—C1	13.31 (12)	C9—C10—C11—C12	−0.4 (3)
Co1—O1—C1—O2	2.2 (2)	C10—C11—C12—C7	−0.7 (3)
Co1—O1—C1—C2	−175.43 (10)	C6—C7—C12—C11	−178.52 (19)
O2—C1—C2—C3	22.5 (2)	C8—C7—C12—C11	1.2 (3)
O1—C1—C2—C3	−159.65 (14)	C15—N1—C13—C14	−0.2 (2)
C1—C2—C3—C4	−106.75 (18)	Co1—N1—C13—C14	177.66 (12)
C1—C2—C3—C8	74.13 (19)	N1—C13—C14—N2	0.2 (2)
C8—C3—C4—C5	1.8 (3)	C15—N2—C14—C13	−0.06 (19)
C2—C3—C4—C5	−177.34 (16)	C16—N2—C14—C13	−179.08 (17)
C3—C4—C5—C6	−0.6 (3)	C13—N1—C15—N2	0.16 (19)
C4—C5—C6—C7	−1.1 (3)	Co1—N1—C15—N2	−177.62 (10)
C5—C6—C7—C12	−178.80 (19)	C14—N2—C15—N1	−0.07 (19)
C5—C6—C7—C8	1.5 (3)	C16—N2—C15—N1	178.96 (16)

Symmetry code: (i) −*x*+1, −*y*+1, −*z*.

### Hydrogen-bond geometry (Å, º)

**Table:**

*D*—H···*A*	*D*—H	H···*A*	*D*···*A*	*D*—H···*A*
O3—H3*A*···O2^ii^	0.84 (2)	2.13 (2)	2.8523 (18)	144 (2)
O3—H3*B*···O2	0.85 (2)	1.81 (2)	2.6463 (16)	168 (2)

Symmetry code: (ii) −*x*, −*y*+1, −*z*.
